# A rapid on-site loop-mediated isothermal amplification technology as an early warning system for the detection of Shiga toxin-producing Escherichia coli in water

**DOI:** 10.1099/mic.0.001485

**Published:** 2024-08-07

**Authors:** Zina Alfahl, Sean Biggins, Owen Higgins, Alexandra Chueiri, Terry J. Smith, Dearbháile Morris, Jean O'Dwyer, Paul D. Hynds, Liam P. Burke, Louise O’Connor

**Affiliations:** 1 Antimicrobial Resistance and Microbial Ecology Group, School of Medicine, University of Galway, Galway, Ireland; 2 Centre for One Health, Ryan Institute, University of Galway, Galway, Ireland; 3 Molecular Diagnostics Research Group, College of Science and Engineering, University of Galway, Galway, Ireland; 4 School of Biological, Earth and Environmental Sciences, University College Cork, Cork, Ireland; 5 Irish Centre for Research in Applied Geosciences (iCRAG), University College Dublin, Dublin, Ireland; 6 Environmental Sustainability and Health Institute, Technological University Dublin, Dublin, Ireland

**Keywords:** *E. coli*, LAMP, STEC, testing, toxins, water contamination

## Abstract

Shiga toxin-producing *Escherichia coli* (STEC) is an important waterborne pathogen capable of causing serious gastrointestinal infections with potentially fatal complications, including haemolytic–uremic syndrome. All STEC serogroups harbour genes that encode at least one Shiga toxin (*stx1* and/or *stx2*), which constitute the primary virulence factors of STEC. Loop-mediated isothermal amplification (LAMP) enables rapid real-time pathogen detection with a high degree of specificity and sensitivity. The aim of this study was to develop and validate an on-site portable diagnostics workstation employing LAMP technology to permit rapid real-time STEC detection in environmental water samples. Water samples (*n*=28) were collected from groundwater wells (*n*=13), rivers (*n*=12), a turlough (*n*=2) and an agricultural drain (*n*=1) from the Corrib catchment in Galway. Water samples (100 ml) were passed through a 0.22 µm filter, and buffer was added to elute captured cells. Following filtration, eluates were tested directly using LAMP assays targeting *stx1*, *stx2* and *E. coli phoA* genes. The portable diagnostics workstation was used in field studies to demonstrate the on-site testing capabilities of the instrument. Real-time PCR assays targeting *stx1* and *stx2* genes were used to confirm the results. The limit of detection for *stx1*, *stx2* and *phoA* LAMP assays were 2, 2 and 6 copies, respectively. Overall, *stx1*, *stx2* and *phoA* genes were detected by LAMP in 15/28 (53.6 %), 9/28 (32.2 %) and 24/28 (85.7 %) samples, respectively. For confirmation, the LAMP results for *stx1* and *stx2* correlated perfectly (100 %) with those obtained using PCR. The portable diagnostics workstation exhibited high sensitivity throughout the on-site operation, and the average time from sample collection to final result was 40 min. We describe a simple, transferable and efficient diagnostic technology for on-site molecular analysis of various water sources. This method allows on-site testing of drinking water, enabling evidence-based decision-making by public health and water management authorities.

## Introduction

*Escherichia coli* (*E. coli*) are common bacteria in the mammalian gastrointestinal tract and part of the normal bacterial flora [1]. While most strains of *E. coli* are harmless, certain *E. coli* strains are pathogenic and can be classified into different pathotypes based on their ability to cause different types of infections and the presence of specific virulence factors. Several categories of pathotypes, collectively termed intestinal pathogenic *E. coli*, cause enteric infections, such as diarrhoea, while extra-intestinal pathogenic *E. coli* pathotypes cause extra-intestinal infections, including urinary tract infections [2]. Some intestinal pathogenic strains produce potent cytotoxins known as Shiga toxins (*stx1* and/or *stx2*) [3], with Shiga toxin-producing *E. coli* (STEC), the most well-known subgroup of the intestinal pathotype enterohaemorrhagic *E. coli*. These toxins play a crucial role in the pathogenesis of diseases caused by STEC such as haemolytic–uremic syndrome and bloody diarrhoea [4]. In Ireland, common STEC serogroups include O157, O26, O103, O111 and O145. These serogroups are of particular concern due to their association with severe clinical outcomes and frequent identification in epidemiological investigations [5].

STEC strains are part of the normal intestinal flora of healthy ruminant animals. These animals, including globally important livestock species, serve as reservoirs for these bacteria, and transmission can occur through contact with contaminated faeces or consumption of contaminated food or water [6]. STEC are of critical concern when present in water sources. One notable example of a STEC outbreak associated with water occurred in May 2000 in Walkerton, Ontario, Canada. The outbreak had severe consequences and was primarily linked to contamination of the town’s water supply, which resulted in widespread illness and several fatalities [7]. The primary source of STEC in water is faecal contamination from animals or infected humans [8]. Agricultural run-off, improper land spreading of manure and contamination from sewage can introduce STEC into water sources. Once present in water, STEC can survive for varying lengths of time, ranging from days to weeks, depending on the environmental conditions [9,10].

Over recent years, Ireland has reported the highest crude incidence rates of STEC enteritis in Europe [11]. These high incidence rates may be associated with livestock-based agricultural practices, including land spreading of manure and poorer drinking water quality in rural areas [12,14]. Contaminated water, especially from private groundwater wells, has been identified as a significant source of STEC infections in Ireland [15]. Individuals relying on private wells for their water supply may face an increased risk of exposure to STEC if the water becomes contaminated, highlighting the importance of proper well maintenance and regular testing [12]. About 720 000 or almost 1 out of every 5 people in Ireland rely on private groundwater supplies for their daily water consumption, in which the majority of these supplies are household wells [16]. Water testing and treatment of private wells are entirely voluntary and fall under the responsibility of the well owner. The guidelines provided by the Environmental Protection Agency (EPA) recommend that all private water supplies undergo testing at least once a year to identify and address microbial contamination. However, the quality of drinking water from private supplies tends to be inferior to that from public supplies, primarily due to the absence of source treatment, less stringent regulation and less frequent monitoring [16].

The primary method for microbiological monitoring of drinking and recreational waters involves enumerating faecal indicator organisms through culture-based techniques [17]. However, this method is associated with numerous limitations including the considerable amount of time required to produce results, typically ranging from 18 to 48 h or more, and the inability to specifically identify STEC which can hinder timely responses in situations where rapid identification is crucial for public health interventions [17]. Bacteriological culture for STEC detection remains the gold standard method, given the importance of identifying viable bacterial isolates for typing. However, culture-based methods are laborious, complex and expensive and exhibit clear limits in sensitivity for STEC detection, especially when dealing with samples containing low concentrations of STEC which can result in false-negative results and an underestimation of the actual contamination concentration [18,19]. Accordingly, a rapid and sensitive test to detect STEC contamination in water is needed to prevent the spread of waterborne illnesses through implementation of timely public health interventions.

Loop-mediated isothermal amplification (LAMP) testing represents a promising and innovative nucleic acid amplification method due to its sensitivity, simplicity and rapid processing [20]. In the current study, we developed and validated an on-site portable diagnostics workstation which enables rapid real-time STEC detection using LAMP technology in 40 min, with proof-of-concept demonstration of the on-site application of the developed workstation performed. Results were evaluated in combination with a previously published rapid water filtration method developed by the Antimicrobial Resistance and Microbial Ecology Group [21]. Proof-of-concept demonstration for the portable on-site application of this technology was performed.

## Methods

**Fig. 1.**
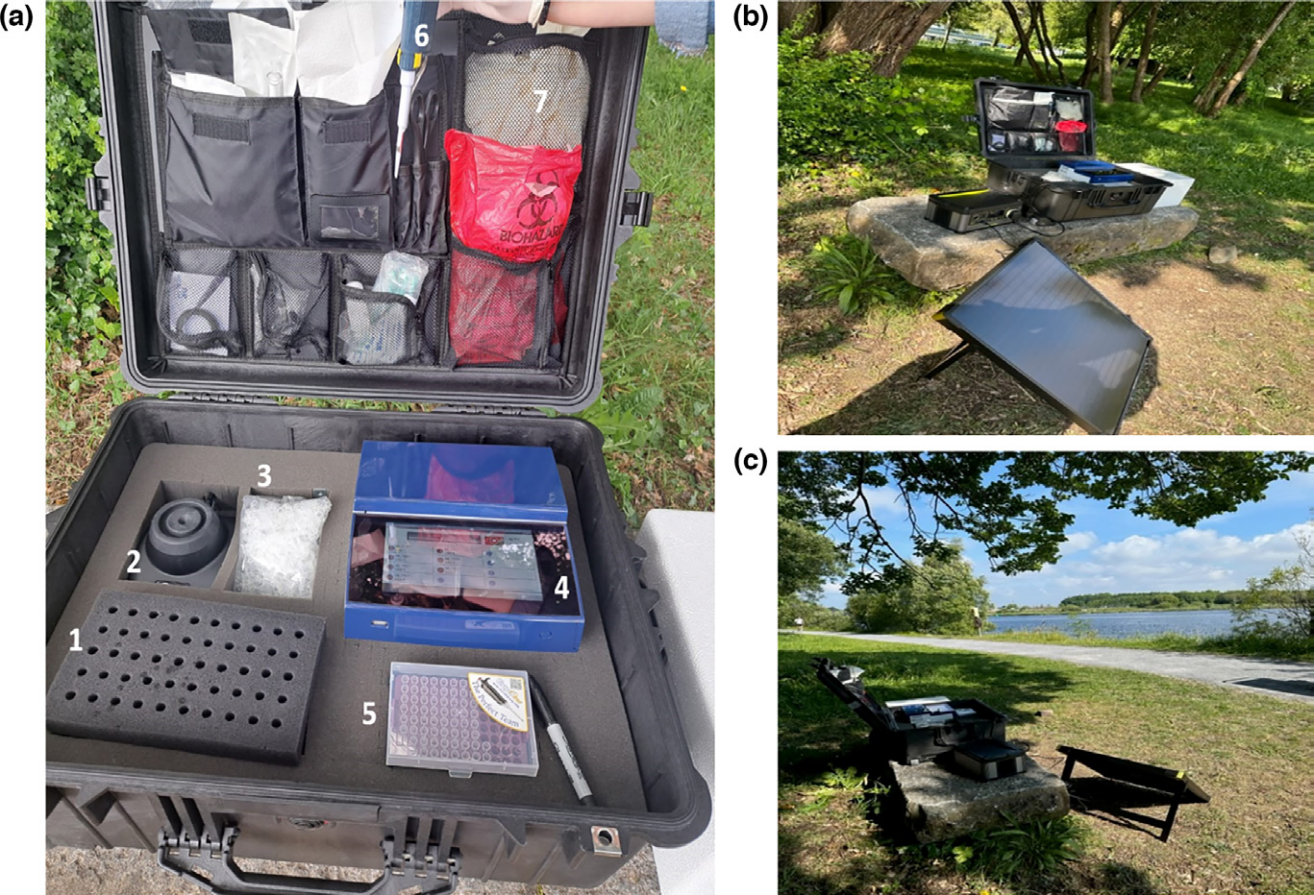
Portable diagnostics workstation. (**a**) Mobile workstation containing (1) microcentrifuge tubes rack, (2) vortex, (3) microcentrifuge tubes, (4) ESEQuant TS2 isothermal nucleic acid amplification instrument, (5) tips, (6) pipette and (7) laboratory consumables. (**b, c**) On-site application.

The on-site LAMP technology consisted of a portable diagnostics workstation housed in a waterproof case (Caulfield Industrial, Ireland) weighing approximately 15 kg. The workstation is composed of ESEQuant TS2 isothermal nucleic acid amplification instrument (Qiagen, Netherlands), vortex, pipette, tips, elution buffer, Sterivex pressure-driven filter unit (Merck Millipore Ltd., Ireland), LAMP reagents [Isothermal Master Mix (OptiGene Ltd., UK), primers and sterile water], Goal Zero power source and disposable sterile consumables (Fig. 1).

**Fig. 2.**
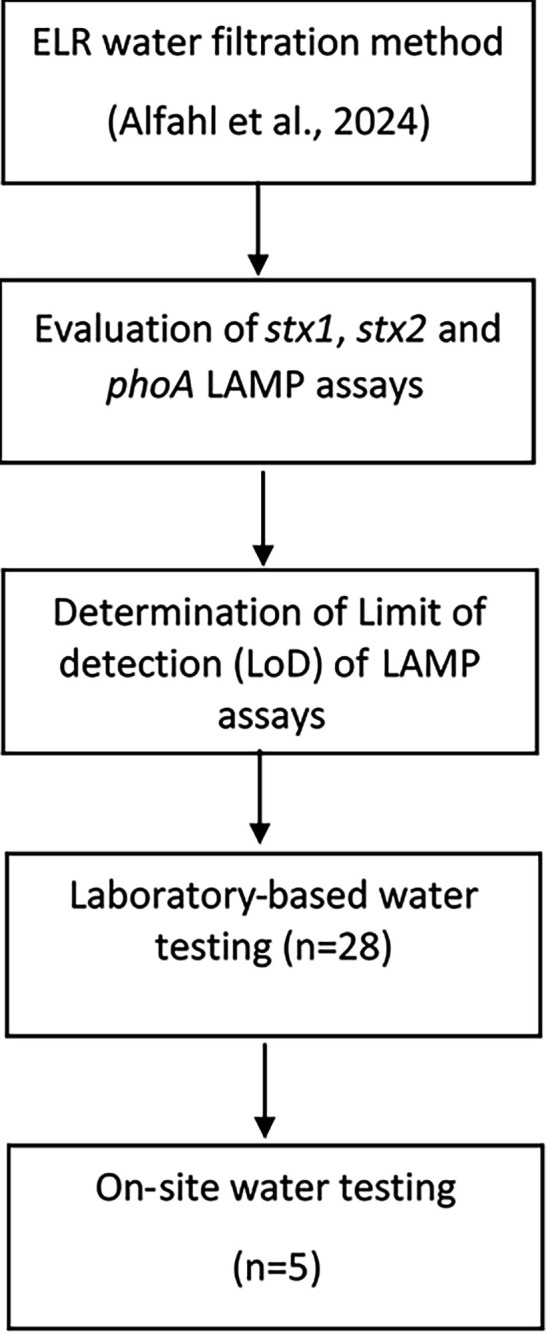
Flowchart summarizing the optimization and validation of the LAMP technology. ELR: enrichment-free, low-volume filtration and rapid lysis method.

The optimization and validation of the LAMP technology were carried out both in the laboratory and on-site as shown in Fig. 2.

### Water filtration

A previously validated novel enrichment-free, low-volume filtration and rapid lysis method (ELR) was used to filter water samples [21]. Briefly, the ELR method comprises passing 100 ml water sample through a 0.22 µm pore size, sterile Sterivex pressure-driven filter unit (Merck Millipore Ltd., Ireland). Following filtration, 400 µl of elution buffer is added to the filter unit, which is vortexed at low speed (speed 7) for 10 min to elute the captured cells from the filter membrane, with the final eluate used for downstream analysis.

### Evaluation of stx1, stx2 and phoA previously published LAMP assays

LAMP assay evaluation was performed in triplicate using the Light Cycler 480 real-time PCR instrument (Roche, Switzerland). Three previously published LAMP assays were evaluated to detect Shiga toxin genes *stx1*, *stx2* and *E. coli* structural genes for alkaline phosphatase, *phoA* [22]. The evaluation was carried out by testing in-house designed gBlocks DNA at varying concentrations (10^8^, 10^3^ and 10^1^ copies; Supplementary A1, Table S1, available in the online Supplementary Material). Positive and negative DNA controls and no template control (NTC) were included in each run. Reaction conditions and primers used are presented in Supplementary A1.

### Determination of limit of detection of LAMP assays

The limit of detection (LoD) of each LAMP assay was determined by preparing known concentrations of *stx1*, *stx2* and *phoA* gBlocks DNA. Eight replicates of concentrations equivalent to 10^8^, 10^7^, 10^6^,10^5^, 10^4^, 10^3^, 10^2^, 10^1^, 8, 6, 4 and 2 copies per reaction were tested in triplicate.

### Statistical analyses

Probit analysis was performed using Minitab 17 statistical software to determine the LoD for LAMP assays using hit rate analysis at 95 % hit rate. Data are presented as numbers and percentages for water samples which were positive for target genes.

### Qualitative real-time PCR for STEC stx1 and stx2 genes

Multiplex PCR was performed in triplicate using the Light Cycler 480 real-time PCR instrument (Roche, Switzerland). A previously published multiplex PCR assay targeting *stx1* and *stx2* genes was used to confirm the results obtained from LAMP [23]. A positive DNA control and a negative DNA control, as well as a NTC, were included in each PCR run. Sample processing, reaction conditions, primers and TaqMan hydrolysis probes used are presented in Supplementary A2.

### Laboratory-based water testing

Water samples were collected from the Corrib catchment in County Galway, West of Ireland. The Corrib catchment is considered a relatively high-risk catchment in terms of STEC risk factors with high laboratory-confirmed human infection rates in 85 % of water samples collected from this area, with 64 % testing positive for the particularly harmful STEC O157 serogroup [24,25]. Water samples (100 ml) (*n*=28) were collected from groundwater wells (*n*=13), rivers (*n*=12), turlough (seasonal lake) (*n*=2) and agricultural drain (*n*=1) sources in sterile containers. To validate the portable diagnostics workstation in the laboratory, eluates were prepared using the ELR method (see “Water filtration” section) and 5 µl was tested using LAMP on the ESEQuant TS2 isothermal nucleic acid amplification instrument and in parallel by PCR under the same cycling conditions using the Light Cycler 480 real-time PCR instrument (Roche, Switzerland).

### On-site water testing

Proof-of-concept for on-site water sample testing in combination with the ELR filtration method was performed with the custom portable diagnostics workstation (Fig. 1). Five locations in the Corrib catchment were visited on a single day, and water samples (*n*=5) were collected from groundwater wells (*n*=3) and rivers (*n*=2). The time from sample collection to final result using the portable diagnostics workstation was approximately 40 min.

## Results

### Validation of the LAMP assays

The three LAMP assays were evaluated in triplicate to detect Shiga toxin genes *stx1* and *stx2* and the *E. coli* structural gene *phoA* by testing in-house designed gBlocks DNA at concentrations (10^8^, 10^3^ and 10^1^ copies). The average time to final results using the Light Cycler 480 real-time PCR instrument was 30 min. Table 1 shows the Cp (crossing point) values for each concentration tested.

**Table 1.** gBlocks DNA concentrations tested with associated Cp values for the validation of the LAMP assays

Concentration (copies/reaction)	Cp values			Average Cp
	**Replicate 1**	**Replicate 2**	**Replicate 3**	
*stx1* gene				
10^8^	5.00	5.00	5.00	5.00
10^3^	7.51	7.58	7.53	7.54
10^1^	8.65	8.67	9.06	8.80
*stx2* gene				
10^8^	5.00	5.00	5.00	5.00
10^3^	10.20	9.54	9.70	9.81
10^1^	14.96	14.81	14.27	14.68
*phoA* gene				
10^8^	5.00	5.00	5.00	5.00
10^3^	9.35	9.11	9.78	9.41
10^1^	11.87	11.53	11.20	11.53

### Determination of the LoD of the LAMP assays

The LoD for the LAMP assays was determined to ensure that the assays are suitable for detecting low copies of target genes *stx1*, *stx2* and *phoA*. Supplementary Table S5 shows the hit rate analysis for the combined data from the three independent runs. The LoD for the *stx1* assay was 2 copies; for the *stx2* assay, it was 2 copies; and for the *phoA* assay, the LoD was 6 copies, indicating that all three assays are highly sensitive in detecting low copies of virulence genes.

### Laboratory-based water sample testing using LAMP

Using LAMP, *stx1*, *stx2* and *phoA* genes were detected in 15/28 (53.6 %), 9/28 (32.2 %) and 24/28 (85.7 %) of water sample eluates, respectively. The LAMP results for *stx1* and *stx2* presence/absence correlated perfectly (100 %) with those obtained using PCR (Table 2) .

**Table 2.** Detection of virulence genes in water samples using LAMP and PCR

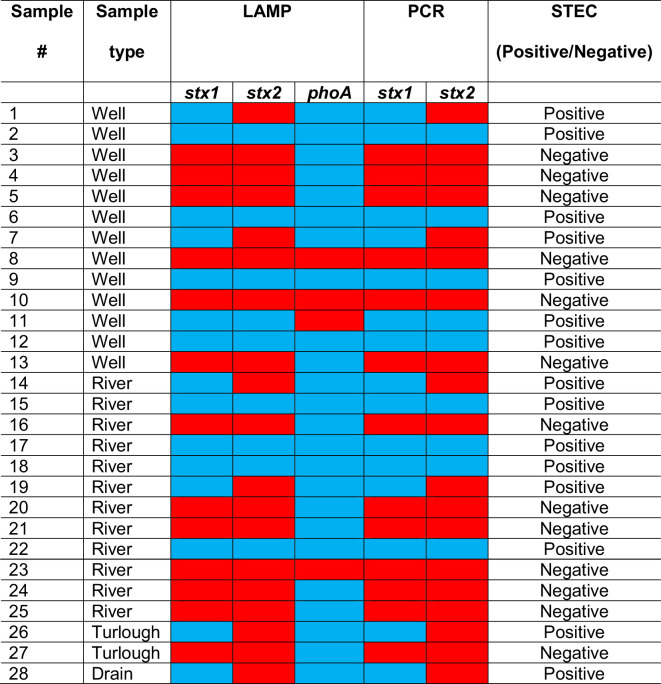 ­

* Blue= gene detected, Red= gene not detected. STEC presence was determined by the presence of at least one Shiga toxin (*stx1* and/or *stx2*).

### On-site water sample testing using LAMP

**Fig. 3.**
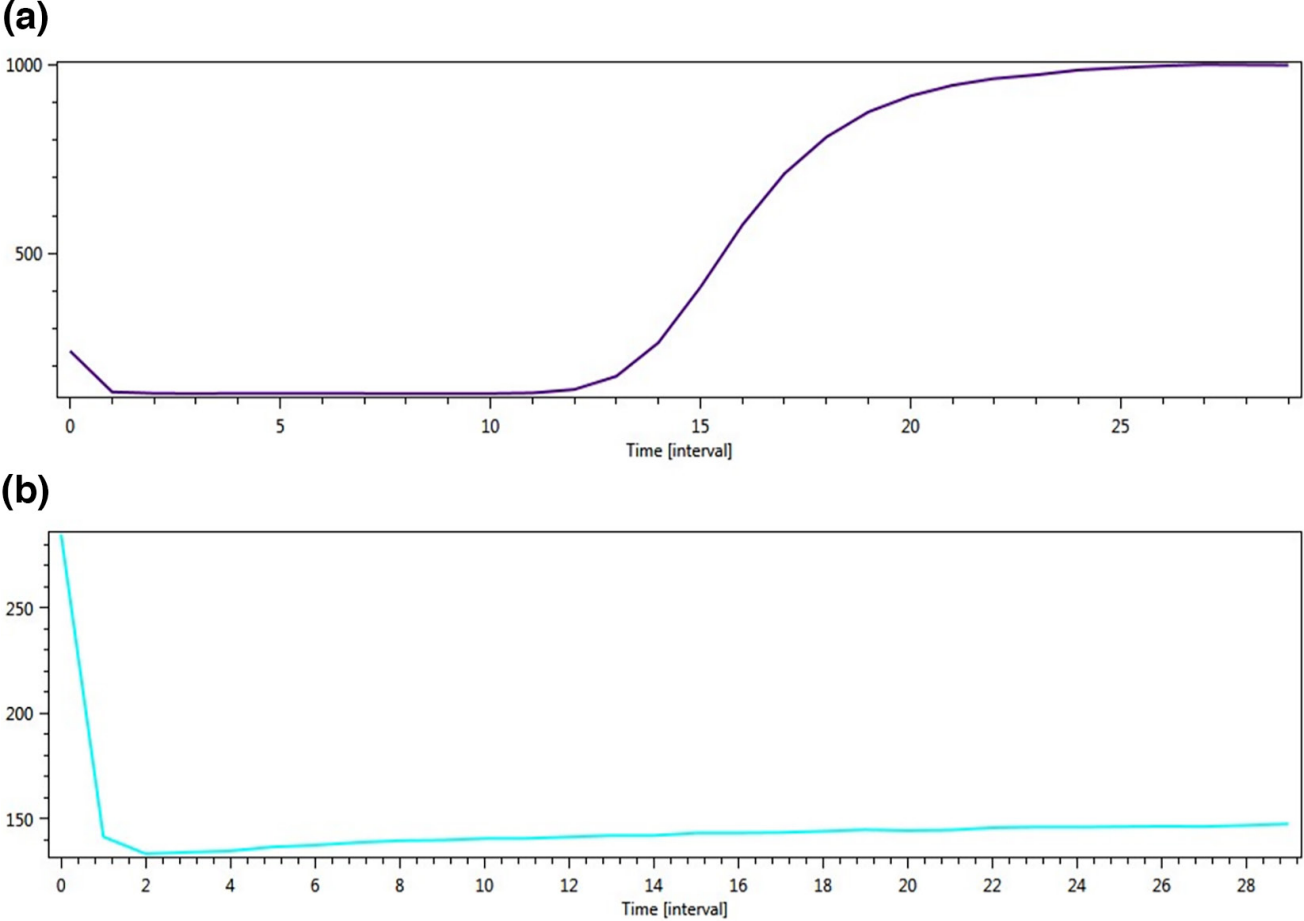
Amplification curves obtained on-site for testing water samples: (**a**) positive sample for target gene and (**b**) negative sample for target gene.

Successful proof-of-concept for the on-site testing of water samples (*n*=5) was demonstrated with the portable diagnostics workstation. The presence or absence of target gene was determined by the generation of an amplification curve which is displayed on the ESEQuant TS2 isothermal nucleic acid amplification instrument screen (Fig. 3). Overall, *stx1*, *stx2* and *phoA* genes were detected in 5/5 (100 %), 3/5 (60 %) and 5/5 (100 %) samples, respectively. The eluates were transported back to the laboratory to confirm results using PCR for *stx1* and *stx2* genes. Table 3 shows the results obtained and the average on-site processing time per sample. The presence of an ‘S’-shaped curve or sigmoid curve confirms a sample is positive for the targeted gene, while the absence of the curve indicates a sample is negative for the targeted gene.

**Table 3.** Detection of virulence genes in water samples using the portable diagnostics workstation

Sample #	Sample type	LAMP			PCR		Processing time
		***stx1***	***stx2***	***phoA***	***stx1***	***stx2***	
1	River	+	+	+	+	+	40 min
2	Well	+	+	+	+	+	40 min
3	River	+	+	+	+	+	40 min
4	Well	+	−	+	+	−	50 min*
5	Well	+	−	+	+	−	

+ =+,gene detected,; −, gene not detected. *Processing time for samples 4 and 5 was higher than the average time per sample as both samples were filtered consecutively and tested in the same run due to proximity of both wells.

## Discussion

The contamination of water with STEC presents a serious public health risk, with potential to cause both waterborne and fresh food-associated foodborne outbreaks. Thus, comprehensive measures to detect, prevent and control the spread of these bacteria in water sources are needed [19,26]. LAMP is a DNA amplification technique with particular appeal for molecular diagnostics which offers several advantages. First, it operates isothermally, eliminating the need for expensive thermocycling equipment. Second, it boasts rapid amplification (processing time=10–20 min) as no temperature ramping is required [27]. Third, LAMP is more tolerant to some inhibitors than PCR [28]. For the detection of STEC in this study, the LAMP assays targeted *stx1* and *stx2* genes as they are considered the main virulence determinants diagnostic for STEC [29]. Furthermore, the LAMP assays targeted the generic *E. coli* (*phoA*) for the detection and differentiation of generic *E. coli* from STEC. Inclusion of *phoA* provides stronger indication of STEC presence/absence, considering that free stx phage particles (bearing *stx1/stx2*) may be present in water samples contaminated with faeces [30].

To date, rapid LAMP tests have been developed for the detection of STEC and other pathogens in different samples such as water, produce, animal and human faecal samples [31,35]. The workstation used in the present study was developed by Higgins *et al.* who used LAMP for the detection of extended-spectrum beta-lactamase enzymes produced by *Enterobacteriaceae* in animal faecal samples [31]. However, to the best of our knowledge, this is the first study that employed a portable workstation using LAMP technology for testing water on-site. Our study showcased real-time detection and differentiation of STEC toxins and virulence genes. In these assays, distinct identification of STEC and *E. coli* was achieved, with each gene being exclusively identified in their respective fluorescence channels. Low-level detection was accomplished in approximately 40 min. Furthermore, the analytical sensitivity analysis of the *stx1*, *stx2* and *phoA* LAMP assays indicated an LOD with 95 % confidence of 2, 2 and 6 copies per reaction, respectively. Studies have demonstrated that LAMP is a highly sensitive method for detecting STEC, with detection limits typically ranging from 10^1^ to 10^3^ c.f.u. ml^−1^ or c.f.u./g, depending on the sample type and protocol. In pure cultures and spiked samples, LAMP can detect STEC at concentrations as low as 10^1^ to 10^2^ c.f.u. ml^−1^ [36,37]. In ground beef, the detection limit is around 10^2^ to 10^3^ c.f.u. g^−1^ after a brief enrichment period, while in faecal samples at concentrations of 10^2^ to 10^3^ c.f.u. g^−1^ [34]. This low LOD indicates greater sensitivity compared with conventional PCR as LAMP has been shown to have 10 times higher sensitivity and amplification efficiency compared with conventional PCR [32,38, 39], allowing for the detection of even small quantities of the target gene. This successful demonstration of robust, rapid and low-level specific differential detection of STEC and *E. coli* marks a significant advancement over existing diagnostic methods for identifying these targets.

Our results highlighted a successful proof-of-concept demonstration for the portable on-site application of the mobile diagnostics workstation combined with the rapid water filtration method with a relatively rapid testing time of approximately 40 min. When compared with current molecular detection methods for STEC used by public health laboratories, which involve multiple steps such as sample transport, filtration, enrichment, DNA extraction and real-time PCR, this LAMP-based method significantly reduces the overall time required for detection. Traditional methods often take several hours to days due to the necessity of enrichment steps and the logistical delays associated with sample transport and processing. In contrast, the rapidity of LAMP allows for near-immediate on-site detection, potentially making it a viable alternative to PCR in diagnostic labs. This method could be particularly useful for preliminary screening, identifying water samples that require further enrichment and cultural isolation, thus streamlining the workflow and enabling faster public health responses to potential STEC contamination in water supplies. We envision the application of this mobile diagnostics workstation as an early warning system to alert public health/environmental health bodies and water managers of possible STEC contamination in water supplies. Rapid detection and intervention are crucial to prevent the spread of STEC-related illnesses and mitigate potential outbreaks. On-site rapid detection allows for judicious use of laboratory resources, flagging potentially contaminated supplies in near real time, which can later be confirmed by culture, if necessary, in the laboratory. The technology is simple to use and interpret and could be utilized by environmental and public health officers or water managers for basic water quality assessments in the field with minimal training. This will result in more engagement within the communities and will educate individuals about the importance of water quality and water testing, and any concerning results could then be escalated to the appropriate public health authorities.

## Conclusions

This study described a simple, transferable and efficient diagnostic technology and demonstrated proof-of-concept in its application to carry out simple on-site analysis of various water sources. Future work should aim to address study limitations by conducting more thorough validation across diverse water types and environmental conditions to ensure robustness and reliability of the method in different real-world scenarios. The detection of STEC in water aligns with the One Health concept, recognizing the interconnectedness of human, animal and environmental health. This method offers the potential to test drinking water on-site allowing swift decisions to be made in relation to usage by local authorities which will contribute to the overall health and well-being of ecosystems, animals and humans.

## supplementary material

10.1099/mic.0.001485 Uncited Supplementary Material 1.

## Abbreviations

E.coli: *Escherichia coli*
EHEC: enterohemorrhagic *E. coli*
ELR: enrichment-free, low-volume filtration and rapid lysis method
EPA: environmental protection agency
ESBL: extended-spectrum beta-lactamase
ExPEC: extra-intestinal pathogenic *E. coli*
FIO: faecal indicator organisms
HUS: hemolytic uremic syndrome
LAMP: loop-mediated isothermal amplification
LoD: limit of detection
NTC: no template control
STEC: Shiga toxin-producing *Escherichia coli*
stx: Shiga toxin
