# Supplementary material for: A rapid on-site loop-mediated isothermal amplification technology as an early warning system for the detection of Shiga toxin-producing Escherichia coli in water
Source: Microbiology (Reading). 2024 Aug 7;170(8):001485. doi: 10.1099/mic.0.001485 (PMC11304963; doi:10.1099/mic.0.001485)
Supplement: Uncited Supplementary Material 1. [file mic-170-01485-s001.pdf]

A rapid on-site Loop-mediated isothermal amplification (LAMP) technology as an early warning system for the detection of Shiga toxin-producing *Escherichia coli* (STEC) in water (Supplementary data)

Zina Alfahl[1,2], Sean Biggins[2,3], Owen Higgins[2,3], Alexandra Chueiri[2,3], Terry J. Smith[2,3], Dearbháile Morris[1,2], Jean O'Dwyer[4,5], Paul D. Hynds[5,6], Liam P. Burke[1,2], Louise O'Connor[2,3*]

[1] Antimicrobial Resistance and Microbial Ecology Group, School of Medicine, University of Galway, Galway, Ireland

[2] Centre for One Health, Ryan Institute, University of Galway, Galway, Ireland

[3] Molecular Diagnostics Research Group, College of Science & Engineering, University of Galway, Ireland

[4] School of Biological, Earth and Environmental Sciences, University College Cork, Cork, Ireland

[5] Irish Centre for Research in Applied Geosciences (iCRAG), University College Dublin, Ireland

[6] Environmental Sustainability and Health Institute, Technological University Dublin, Ireland

*Corresponding authors at:

Molecular Diagnostics Research Group, College of Science & Engineering, University of Galway, Ireland. Email address: louise.oconnor@universityofgalway.ie (L.O'Connor)

A1. LAMP assays

Table S1 gBlocks® DNA sequences for *stx1*, *stx2* and *phoA* genes

Name	Sequence 5' – 3'
stx1 gBlock®	ACAACAGCGGTTACATTGTCTGGTGACAGTAGCTATACCACGTTACAG<br>CGTGTTGCAGGGATCAGTCGTACGGGGATGCAGATAAATCGCCATTCG<br>TTGACTACTTCTTATCTGGATTTAATGTCGCATAGTGGAACCTCACTGACG<br>CAGTCTGTGGCAAGAGCGATGTTACGGTTTGTTACTGTGACAGCTGAAGCT<br>TTACGTTTTCGGCAAATACAGAGGGGATTTCGTACAACACTGGATGATC
stx2 gBlock®	GCATCCAGAGCAGTTCTGCGTTTTGTCACTGTCACAGCAGAAGCCTTACGCTTCAGG<br>CAGATACAGAGAGAATTTCGTCAGGCACTGTCTGAAACTGCTCCTGTGTATACG<br>ATGACGCCGGGAGACGTGGACCTCACTCTGAACTGGGGGCGAATCAGCA<br>ATGTGCTTCCGGAGTATCGGGGAGAGGATGGTGTCAGAGTGGGGAGAA<br>TATCCTTTAATAATATATCAGCGATACTGGGGACTGT GGCCGTTATACTG
phoA gBlock®	TTTGTTACTGTGACAGCTGAAGCTTTACGTTTTCGGCAAATACAGAG<br>GGGATTTCGTACAACACTGGATGATCTCAGTGGGCGTTCTTATGTAATGACTGCTGAAG<br>ATGTTGATCTTACATTGAACTGGGG

LAMP assays primer set used in this study consisted of two outer (F3, B3), two inner (FIP, BIP) and two loop primers (Loop F, Loop B), which recognised eight different regions of the gene target (Stratakos et al., 2017). Table S2 shows the primers used for each LAMP assay. The final reaction mix volume for each reaction contained 15 µL Isothermal Master Mix (OptiGene Ltd., UK), 2 µL of 100 µM primers mix, 3 µL molecular grade water and 5 µL of target template eluate bringing the total reaction volume to 25 µL.

For each assay the positive control was the gBlock® DNA for each target (Table S1) at 10^3 copies/µL. No template control (NTC) was used as a negative control.

The LAMP assay was run at 63°C in ESEQuant TS2 isothermal nucleic acid amplification instrument (Qiagen, Netherlands) for 30 min.

Table S2 Primers used for LAMP assays

	Name	Sequence (5' to 3')
stx1	FIP	GCG ATT TAT CTG CAT ATG TCT GGT AGT AGC TAT
	BIP	GGA ACC TCA CTG ACG CAG TCC TTC AGC TGT CAC AGT AAC A
	LF	ACT GAT CCC TGC AAC ACG
	LB	TGT GGC AAG AGC GAT GTT
	F3	ACA ACA GCG GTT ACA TTG T
	B3	GAT CAT CCA GTG TTG TAC GAA
stx2	FIP	GGC GTC ATC GTA TAC ACA GGA GCG CAG GCA GAT ACA G
	BIP	AGA CGT GGA CCT CAC TCT GAA ACT CTG ACA CCA TCC TCT C
	LF	CAG ACA GTG CCT GAC GAA
	LB	GGC GAA TCA GCA ATG TGC
	F3	GCA TCC AGA GCA GTT CTG
	B3	CAG TAT AAC GGC CAC AGT C
phoA	FIP	GTG ATC AGC GGT GAC TAT GAC CTC TCG ATG AAG CCG TAC A
	BIP	ATT GTC GCG CCG GAT ACC CTC ATC ACC ATC ACT GCG
	LF	AGC GTG TTG CCA TCC TTT
	LB	CAG GCG CTA AAT ACC AAA GAT G
	F3	AAG TTG AAG GTG CGT CAA T
	B3	CTT GTG AAT CCT CTT CGG AG

A2. Multiplex Real time PCR

Multiplex PCR assay for detection of virulence *stx1* and *stx2* genes was performed as previously described (Perelle et al., 2004). Table S3 shows the primers and probes used.

gBlocks® DNA (10^3 copies/µL) of each target were used as a positive control (Table S4). No template control (NTC) was used as a negative control.

The cycling conditions were as follows: pre-incubation at 50 °C for 2 min and 95 °C for 20 s, followed by 40 cycles (95 °C for 3 s and 60 °C for 30 s). Data analysis was performed using the real time Light Cycler PCR software.

Table S3 Primers and Probes used for *stx1* and *stx2* PCR assays

Gene	Name	Sequence (5' to 3')
stx1	Forward primer	TTT GTY ACT GTS ACA GCW GAA GCY TTA CG
	Reverse primer	CCC CAG TTC ARW GTR AGR TCM ACR TC
	Probe	FAM-CTG GAT GAT CTC AGT GGG CGT TCT TAT GTA A-BHQ1
stx2	Forward primer	TTT GTY ACT GTS ACA GCW GAA GCY TTA CG
	Reverse primer	CCC CAG TTC ARW GTR AGR TCM ACR TC
	Probe	CY5-TCG TCA GGC ACT GTC TGA AAC TGC TCC-BHQ2

Table S4 PCR assays positive controls

	**Sequence 5' – 3'**
stx1 gBlock®	TTTGTTACTGTGACAGCTGAAGCTTTACGTTTTCGGCAA ATACAGAGGGGATTTCGTACAACACTGGATGATCTCAGT GGGCGTTCTTATGTAATGACTGCTGAAGATGTTGATCTT ACATTGAACTGGGG
stx2 gBlock®	GCATCCAGAGCAGTTCTGCGTTTTGTCACTGTCACAGCA GAAGCCTTACGCTTCAGGCAGATACAGAGAGAATTTCGT CAGGCACTGTCTGAAACTGCTCCTGTGTATACGATGACG CCGGGAGACGTGGACCTCACTCTGAACTGGGGGCGAAT CAGCAATGTGCTTCCGGAGTATCGGGGAGAGGATGGTG TCAGAGTGGGGAGAATATCCTTTAATAATATATCAGCGA TACTGGGGACTGTGGCCGTTATACTG

Table S5 Hit rate analysis for *stx1*, *stx2* and *phoA* LAMP assays

	**Target input genome copies**	**Number of replicates tested**	**Number of replicates detected in PCR assay**
stx1	10^8	24	24
	10^7	24	24
	10^6	24	24
	10^5	24	24
	10^4	24	24
	10^3	24	24
	10^2	24	24
	10^1	24	24
	8	24	24
	6	24	24
	2	24	24
stx2	10^8	24	24
	10^7	24	24
	10^6	24	24
	10^5	24	24

	10^4	24	24
	10^3	24	24
	10^2	24	24
	10^1	24	24
	8	24	24
	6	24	24
	2	24	24
phoA	10^8	24	24
	10^7	24	24
	10^6	24	24
	10^5	24	24
	10^4	24	24
	10^3	24	24
	10^2	24	24
	10^1	24	24
	8	24	24
	6	24	21
	2	24	15

References

Perelle S, Dilasser F, Grout J, Fach P. Detection by 5'-nuclease PCR of Shiga-toxin producing Escherichia coli O26, O55, O91, O103, O111, O113, O145 and O157:H7, associated with the world's most frequent clinical cases. Mol Cell Probes 2004; 18: 185-92.

Stratakos AC, Linton M, Millington S, Grant IR. A loop-mediated isothermal amplification method for rapid direct detection and differentiation of nonpathogenic and verocytotoxigenic Escherichia coli in beef and bovine faeces. J Appl Microbiol 2017; 122: 817-828.
